# Genomic differences between black and white patients implicate a distinct immune response to papillary renal cell carcinoma

**DOI:** 10.18632/oncotarget.14122

**Published:** 2016-12-23

**Authors:** David J. Paulucci, John P. Sfakianos, Anders J. Skanderup, Kathleen Kan, Che-Kai Tsao, Matthew D. Galsky, A. Ari Hakimi, Ketan K. Badani

**Affiliations:** ^1^ Department of Urology, Icahn School of Medicine at Mount Sinai Hospital, New York, NY, USA; ^2^ Computational Biology Program, Sloan Kettering Institute for Cancer Research, Memorial Sloan Kettering Cancer Center, New York, NY, USA; ^3^ Department of Medicine, Division of Hematology/Oncology, Tisch Cancer Institute, Icahn School of Medicine at Mount Sinai, New York, NY, USA; ^4^ Department of Surgery—Urology Service, Memorial Sloan Kettering Cancer Center, New York, NY, USA

**Keywords:** papillary renal cell carcinoma, racial disparities, immune system signaling, targeted therapy, immune response

## Abstract

Significant disparities in survival, incidence and possibly response to current therapies exist between black and white patients with renal cell carcinoma (RCC). Recent genomic evidence to account for these disparities has been reported for clear cell RCC. However, racial disparities at the genomic level for papillary RCC (pRCC) which is a genetically distinct and less responsive histologic subtype of RCC have not been reported. Using The Cancer Genome Atlas (TCGA) data, the present study assessed gene-level expression, somatic mutation and pathway differences between 58 black and 58 white patients with pRCC propensity matched on age, gender and pathologic T stage. Distinct tumor biology with differential expression patterns were observed in black vs. white patients with pRCC. Specifically, significance analysis of microarrays was applied to TCGA gene expression data and identified 163 genes and 120 genes overexpressed in black and white patients, respectively (FDR q<0.05). Gene Set Enrichment Analysis identified 62 gene sets enriched (p<0.10) in blacks. Enrichment of immune immune system pathways were noted in black patients. These included the B cell receptor signaling pathway, the NOD-like receptor signaling pathway and genes involved in defensins. The VEGF pathway was also more significant in black patients. CRYBB2, a gene associated with the WNT pathway was overexpressed in Black patients. While our data requires validation, these findings suggest that race may have implications for distinct immune responses to cancer and that the use of immunotherapies, and VEGFR inhibitors to target these pathways may improve survival in black patients with advanced pRCC.

## INTRODUCTION

The 5 year survival rate for the estimated 61,560 new cases of kidney cancer in 2015 is 73% [1]. Survival from kidney cancer is heavily dependent on the stage of disease with a 5 year survival rate of 12% for patients with metastatic RCC [1]. Strong evidence also exists to suggest that survival from RCC is dependent on race with studies showing worse 5 year overall survival for black vs. white patients (68.0% vs. 72.6%), despite black patients being more likely to present with localized RCC [2–7]. Specifically in a recent study by Rose et al. using the National Cancer Database, it was found that black compared to white patients with stage IV RCC before and during the targeted therapy era had worse survival irrespective of age, comorbidities, income, insurance, treatment facility type, grade, histology, receipt of nephrectomy and receipt of systemic therapy [7].

While lack of access to quality health care, lower rates of nephrectomy, greater use of alcohol, tobacco and higher rates of obesity and hypertension are suggested to underlie disparities in survival and incidence between black and white patients [3, 4, 6, 8], recent reports have suggested that differences in tumor biology of RCC may also contribute to disparities in survival between black and white patients [7, 9]. Particularly in a study of black and white patients with clear cell RCC (ccRCC) by Krishnan et al. using both The Cancer Genome Atlas (TCGA) data set and a validation set, it was found that VHL mutations occurred at a lower frequency in black patients and also that vascular endothelial growth factors (VEGF) and hypoxia-inducible factor (HIF) pathways were up-regulated less in black patients [9].

Racial disparities in survival also appear to be regardless of histology as evidenced by worse survival for black patients in the study by Rose et al in a predominantly ccRCC cohort and by Pai et al. in a predominantly pRCC cohort [7, 10]. While the study by Krishnan et al. offers strong genomic evidence as to why survival is worse in black patients despite the proliferation of VEGF-targeted therapies, it is limited to ccRCC and includes no patients with papillary RCC (pRCC) [9].

No studies have characterized genomic differences between black and white patients with pRCC; a genetically and phenotypically distinct form of RCC that occurs at a higher rate in black patients [2]. pRCC vs. ccRCC is specifically characterized by MET mutations and gains of chromosomes 7,12,16 and 17 as possible drivers [11, 12] whereas losses of heterozygosity of chromosome 3p and inactivating mutations of the VHL gene characterize ccRCC [13]. Additionally, while pRCC occurs less frequently than ccRCC [2] and is also less likely to metastasize than ccRCC [14], pRCC vs. ccRCC when in the presence of vena cava thrombus is worse [15] and yields lower response rates to current targeted molecular therapies (e.g., sunitinib, temsirolimus) [16, 17].

The current study therefore sought to identify gene-level expression, pathway and non-silent somatic mutation differences between black and white patients with pRCC.

## RESULTS

### Demographic, clinical, pathologic outcomes and survival

Demographic, clinical and pathologic features for the pre and post propensity matched cohorts are presented in Table 1. Among the 58 black patients and 58 white patients in post-propensity score matched cohort, no differences were found in any demographic, clinical or pathologic features including age (p=.536) and pathologic stage (p=.937).

**Table 1:** Clinical Characteristics and Pathologic Features between Black and White pRCC Patients

	*Pre-Propensity Matched Cohort*			*Propensity Matched Cohort*		
	*Black Patients*	*White Patients*	*P Value*	*Black Patients*	*White Patients*	*P Value*
Patients	58 (22.8%)	196 (77.2%)		58 (50.0%)	58 (50.0%)	
Age	59.0 (50.0-66.5)	63.0 (55.0-71.0)	**.018**	59.0 (50.0-66.5)	59.5 (52.3-66.8)	.536
Male	37 (63.8%)	145 (74.0%)	.131	37 (63.8%)	44 (75.9%)	.157
Hispanic or Latino	1(1.9%)	9 (5.1%)	.460	1 (1.9%)	1 (1.8%)	>.999
BMI	30.4 (25.5-33.8)	28.0 (25.5-31.8)	.115	30.4 (25.5-33.8)	27.4 (25.1-29.5)	**.029**
Karnofsky Performance Score	90 (90-100)	100 (90-100)	.477	90 (90-100)	100 (90-100)	.658
USA Case	48 (100.0%)	152 (89.9%)	**.016**	48 (100.0%)	41 (82.0%)	**.003**
Subtype						
Type 1	20 (60.6%)	51 (44.7%)	.108	20 (60.6%)	18 (50.0%)	.377
Type 2	13 (39.4%)	63 (55.3%)		13 (39.4%)	18 (50.0%)	
Year of Initial Pathologic Diagnosis	2011 (2009-2012)	2010 (2008-2012)	.086	2011 (2009-2012)	2010 (2008-2012)	.148
Smoking Status			.544			.376
Never	21 (40.4%)	80 (49.1%)		21 (40.4%)	21 (44.7%)	
Former	22 (42.3%)	60 (36.8%)		22 (42.3%)	13 (29.8%)	
Current	9 (17.3%)	23 (14.1%)		9 (17.3%)	12 (25.5%)	
Tumor Laterality			.103			.469
Right	28 (49.1%)	85 (43.4%)		28 (49.1%)	23 (39.7%)	
Left	27 (47.4%)	110 (56.1%)		27 (47.4%)	34 (58.6%)	
Bilateral	2 (3.5%)	1 (0.5%)		2 (3.5%)	1 (1.7%)	
Clinical						
T1	36 (72.0%)	96 (73.3%)		36 (72.0%)	25 (69.4%)	
T2	10 (20.0%)	15 (11.5%)	.186	10 (20.0%)	8 (22.2%)	.937
T3	4 (8.0%)	20 (15.3%)		4 (8.0%)	3 (8.3%)	
N1	3 (5.2%)	10 (7.5%)	.294	3 (5.2%)	4 (6.9%)	.572
Pathologic						
Tumor Size	4.5 (3.0-6.5)	4.0 (2.8-6.0)	.292	4.5 (3.0-6.5)	4.8 (2.7-8.5)	.677
Tumor Weight	208.5 (120.8-350.0)	200 (136.5-300.0)	.763	208.5 (120.8-350.0)	200.0 (160.0-323.0)	.933
T1	40 (69.0%)	137 (69.9%)		40 (69.0%)	36 (62.1%)	
T2	12 (20.7%)	16 (8.2%)	.009	12 (20.7%)	13 (22.4%)	.654
T3	6 (10.3%)	43 (21.9%)		6 (10.3%)	9 (15.5%)	
N1	3 (5.2%)	16 (8.2%)	.578	3 (5.2%)	4 (6.9%)	>.999
Tumor Necrosis Present	18 (31.2%)	77 (39.3%)	.290	18 (31.2%)	28 (48.3%)	.068
Percent of Tumor Nuclei Present	85% (70-90%)	85% (80-90%)	.788	85% (70-90%)	87% (80-90%)	.608

For categorical variables, chi square tests performed. Frequencies presented with percentages in parenthesis.

For continuous variables, Mann- Whitney U tests performed. Medians presented with inter-quartile range in parenthesis.

In the post-propensity matched cohort, black and white patients had no differences in overall survival (HR=0.47, p=.336) and cancer-specific survival (HR=1.00, p=.999).

### Supervised whole genome expression analysis

**Figure 1:**
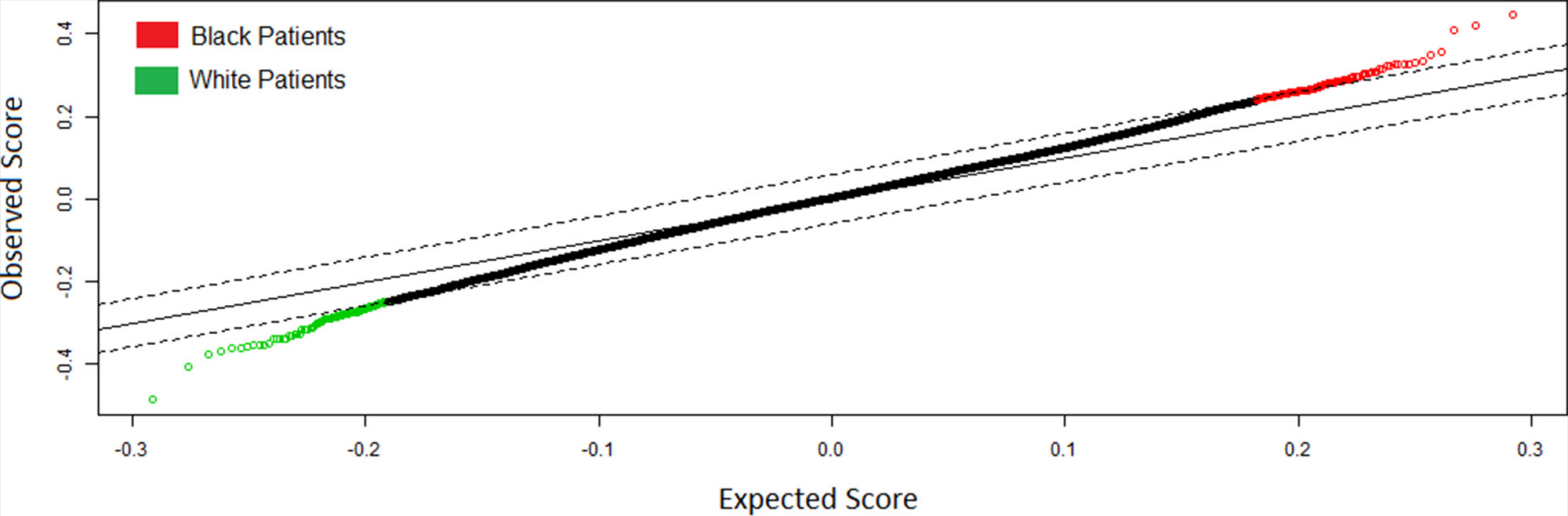
Significance Analysis of Microarrays Plot of Genes Differentially Expressed (q < .05) Between Black and White Patients with Papillary Renal Cell Carcinoma

Significance analysis of Microarrays (SAM) analysis identified 283 differentially expressed genes after false discovery rate correction (FDR q<.05) (Figure 1). 163 genes were overexpressed in black patients and 120 genes were overexpressed in white patients. For the propensity matched cohort, all genes differentially expressed with corresponding 2-fold or greater change are presented in Table 2. All genes differentially expressed can be identified in Supplementary Table S1. Genes overexpressed in black vs. white patients with a fold change ≥ 2 included DHX40P1 (Fold Change (FC) = 2.46 E8), ATCAY (FC=2.46 E8), TREML4 (FC=2.25 E8), LOC100124692 (FC=1.52 E8), GSTM1 (FC=42.33), FCN2 (FC=4.92), GRIN21 (FC=4.76), FAM153A (FC=4.66), UBD (FC=4.38), CRYBB2 (FC=4.37), FLT3 (FC=3.93), FAM70A (FC=3.41), MGAM (FC=3.35), LRRC55 (FC=3.33), CCL3L11 (FC=3.12), SOX30 (FC=2.84), JAKMIP1 (FC=2.82) and GSTT2 (FC=2.75) among others.

**Table 2:** Genes Differentially Overexpressed in Black Compared to White pRCC Patients with a Fold-Change ≥ 2

Gene Overexpressed	Fold-Change	q-value
DHX40P1	2.46 E8	0.011
ATCAY	2.46 E8	0.031
TREML4	2.25 E8	0.036
LOC100124692	1.52 E8	0.036
GSTM1	42.23	0.05
FCN2	4.92	0
GRIN2A	4.76	0.031
FAM153A	4.66	0.043
UBD	4.38	0
CRYBB2	4.37	0
FLT3	3.93	0
FAM70A	3.41	0.018
MGAM	3.35	0.05
LRRC55	3.33	0
CCL3L1	3.12	0.026
SOX30	2.84	0.018
JAKMIP1	2.82	0.041
GSTT2	2.75	0.036
PRSS45	2.57	0.031
GRAP2	2.37	0.045
EMR1	2.34	0
CA8	2.28	0.031
CXCL9	2.27	0.018
TARP	2.22	0.043
IRF4	2.22	0.043
CXCL10	2.15	0.036
HLA-DPB2	2.11	0.036
CPT1C	2.11	0.05
P2RY10	2.1	0.046
LPL	2.08	0.05
CHST1	2.07	0
PDE2A	2.07	0
SCUBE1	2.02	0.047
FAM162B	2.01	0.011

* No overexpressed genes in White patients had a fold change ≥ 2.

### Pathway analysis

**Figure 2:**
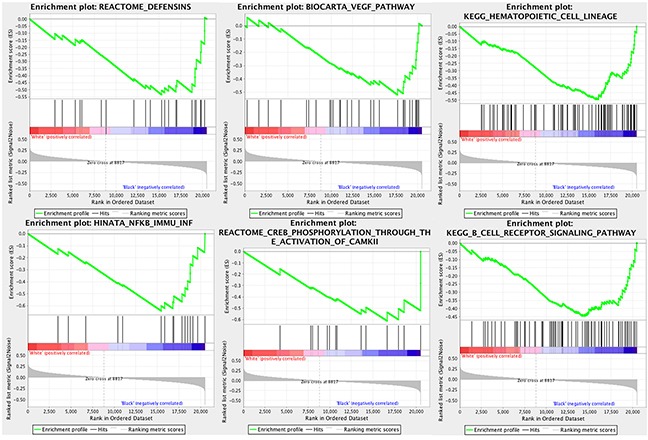
Gene sets overrepresented in black patients with pRCC −log10 of p-values are shown in the different subsets.

We identified 62 genes sets that were enriched in black patients (nominal p<0.10). The complete list of gene sets is shown in Supplementary Table S2. GSEA identified several immune related pathways enriched in black patients including the B cell receptor signaling pathway (p=.097), NOD-like receptor signaling pathway (p=.054), and genes involved in defensins (p=.042) (Figure 2). Furthermore, enrichment of the VEGF pathway; p=0.040, was identified in black patients (Figure 2).

### Gene-level mutations

Results from gene-level somatic mutation rate comparisons between black and white patients are presented in Table 3. MUC4 was mutated at a higher rate in black patients (26.7% vs. 7.3%, p=.044) and PCDHGC5 was mutated at a higher rate in white patients (17.1% vs. 0.0%, p=.018).

**Table 3:** Comparison of Gene-Level Non Silent Somatic Mutations Between Black and White Papillary RCC Patients

Gene	*Black Patients*	*White Patients*	*P Value*
abParts	10 (24.4%)	4 (13.3%)	.247
DDX12P	5 (12.2%)	6 (20.0%)	.509
FLJ36000	5 (12.2%)	5 (16.7%)	.733
FRG1B	2 (4.9%)	3 (10.0%)	.644
CROCCP2	5 (12.2%)	4 (13.3%)	>.999
MUC4	3 (7.3%)	8 (26.7%)	**.044**
MGC70870	13 (31.7%)	6 (20.0%)	.271
MST1P2	5 (12.2%)	5 (16.7%)	.733
PCDHGC5	7 (17.1%)	0 (0.0%)	**.018**
TTN	4 (9.8%)	5 (16.7%)	.479

Chi square tests of Independence of Fisher's Exact Tests performed. Frequencies presented with percentages in parenthesis.

## DISCUSSION

Previous studies have shown that survival for black vs. white patients with RCC is worse [7, 10]. While previous reports suggest that factors including a higher prevalence of hypertension, smoking, alcohol use and lack of access to care underlie this survival disparity [3, 4, 6, 8], this survival disparity may also be explained by the present study's identification of distinct tumor biology between black and white patients with papillary RCC [9]. Specifically in the present study's supervised whole genome expression analysis between 58 black and 58 white propensity score matched patients with pRCC, we identified a distinct tumor biology as demonstrated by enrichment of immune related pathways including the B cell receptor signaling pathway, NOD-like receptor signaling pathway, and genes involved in defensins, VEGF and more specifically overexpression of CRYBB2 associated with the WNT signaling pathway in black patients.

The VEGF pathway in particular plays a prominent role in angiogenesis, resistence to therapy and metastases [18–21]. Thus, enrichment of this pathway in black vs. white patients may contribute to worse survival observed in black patients with RCC. Additionally, overexpression of CRYBB2 which is associated with the WNT signaling pathway involved in tumor progression, growth, differentiation and metastases [22, 23] may also contribute to worse survival observed in black patients with RCC.

Our data raises questions regarding racial differences in cancer biology. This is important to consider when executing clinical trials. In RCC trials this is a significant issue. In a study of Nivolumab vs. Everolimus for ccRCC, only 6 (0.6%) of the 821 patients included were black [24]. Similarly in a randomized controlled trial comparing Cabozantinib and Everolimus for advanced RCC, 658 patients were included in the study of which 532 (80.1%) patients were white only 9 (1.4%) patients were black [25]. Also in a randomized controlled trial Pazopanib vs. placebo of 435 patients, 1 (0.2%) patient was black and 374 (85.9%) patients were white [26]. Among the studies of targeted therapy and immunotherapy in pRCC in which race is reported, 11% (n=12/108) is the highest proportion of black patients reported for any study [27, 28]. With our demonstration of distinct pRCC biology and Krishnan et al.'s demonstration of distinct ccRCC biology between black and white patients, it is likely inaccurate to apply findings within predominantly white cohorts to black patients with RCC for either histologic subtype. Additionally, efforts should be made to ensure that trials are set up in geographically accessible areas and that black patients with RCC are made aware of these trials.

Enrichment of the VEGF pathway may predict increased responsiveness of black patients with advanced pRCC to VEGF receptor (VEGFR) inhibitors. Currently, positive upstream regulators of the PI3K/AKT/mTOR signaling pathway including VEGFRs and their ligands are primary targets in the treatment of advanced RCC using VEGFR and mTOR inhibitors including sunitinib, temsirolimus, everolimus, bevacizumab and sorafenib among others [29, 30]. However, activity for these TMTs is lower for advanced pRCC vs. ccRCC [16, 17] and the NCCN guidelines recommend clinical trial enrollment as the preferred treatment option for patients with advanced pRCC [31]. Specifically in the treatment of locally advanced or metastatic pRCC, progression-free survival for sunitinib, everolimus and temsirolimus ranged from 1.6-8.1 months, 3.4-5.5 months and 5.9 months, respectively. [27–28, 32–36].

While results from these studies demonstrate poor efficacy of VEGFR and mTOR inhibitors for advanced pRCC overall, our finding of VEGF enrichment in black patients suggests increased responsiveness of these therapies particularly in black patients with advanced pRCC. Distinct tumor biology has previously been identified in ccRCC; particularly with less up regulation of HIF and VEGF pathways in black patients to suggest a lower response to VEGF targeted therapies among black patients with ccRCC. However, pRCC is a genetically distinct form of RCC driven by MET mutations and gains of chromosomes 7,12,16 and 17 as possible drivers [11, 12]; and in the present study, enrichment of VEGF pathway conversely suggests increased responsiveness of VEGFR tartgeted therapies among black patients with pRCC.

Increased responsiveness in black patients with the use of targeted therapies for these pathways should be validated and the use of VEGFR therapies in the adjuvant or first line setting should be explored in future clinical trials for advanced pRCC. Additionally, while the number of patients in randomized trials for pRCC vs. ccRCC is already low and the number of black vs. white patients in trials for RCC is even lower, multi-institutional efforts to pool data should be undertaken to analyze whether trials utilizing VEGFR therapies are producing a favorable response in black patients with advanced pRCC. Such efforts would serve as a route to reduce survival disparities between black and white patients with RCC.

In addition, enrichment of immune related pathways involved in B cell receptor signaling, NOD-like receptor signaling, genes involved in defensins and more specifically overexpression of genes involved in immune-related pathways and processes (RHOH, TREML4, FCN2, CCL3L1, GRAP2, CXCL9, TARP, IRF4, CXCL10, CXCL11, IFITM1, LAMP3, GATA2, etc.) were found in black patients. To further understand the relationship between increased immune activity in tumors of black patients and racial outcome disparities in RCC, future studies are needed. An increased immune response to pRCC in black vs. white patients would suggest that enrichment of the VEGF pathway and overexpression of genes involved in the WNT pathway in addition to other pathologic factors (i.e., hypertension, smoking status, access to care) are underlying worse outcomes for black patients with RCC. Nonetheless, overactivity of immune related pathways in black patients implicates a greater response and larger role for immunotherapies in the first line or adjuvant setting for these patients.

Nivolumab (a PD-1 immune checkpoint inhibitor) compared to Everolimus (an mTOR inhibitor) has recently been shown to result in a greater objective response rate (25% vs. 5%) and longer overall survival in ccRCC; however no patients enrolled had pRCC [24]. In a recent case report by Geynisman et al. a rapid response to Nivolumab was observed in a patient with metastatic pRCC with sarcomatoid and rhaboid features [36]. While this patient only received Nivolumab due to his declining performance status and development of subcutaneous lesions, future studies of Nivolumab in the first-line setting or adjuvant setting should enroll patients with pRCC as a favorable response may be observed overall and particularly in black patients.

Additionally, a study of interferon-alfa showed progression-free survival for interferon to be 2.1 months for pRCC [37]. While the use of interferon for pRCC evidently yields poor oncologic outcome, its use for advanced pRCC should be further explored as should the use of interferon before VEGF therapies which is being explored for ccRCC [38] since black patients may be more likely to experience an oncologic benefit. Multi-institutional efforts to pool data from previous and current trials of pRCC should also be undertaken to assess whether there is increased efficacy of immunotherapies including interferon and IL-2 in black patients with advanced pRCC. Such studies would allow for a better understanding of the clinical efficacy of these drugs in black patients for future studies.

CRYBB2 associated with the WNT pathway was overexpressed in Black patients and other studies in colon and breast cancer have shown promising results for targeting of the WNT pathway with small molecules, peptides and blocking antibodies [23].

Although we propensity score matched black and white patients on age, gender and pathologic T stage, these findings are limited by unavailable/missing data and a relatively small sample size. Specifically, lack of data within the TCGA on hypertension, alcohol use, BMI (n=65) and pRCC subtype (n=107) limit us from attributing these gene-level expression differences to race alone. Additionally, the limited sample size of this study and the lack of a validation data set confounds the generalizability of these findings and highlights the need for independent validation of these results for reliability and validity.

An additional limiting factor is that we are unable to show a survival difference in this study, perhaps due to the low sample size. While the majority of patients in the study by Pai et al. showing worse survival for black patients had pRCC, this study was not exclusive to pRCC and included several patients with chromophobe and collecting duct RCC [10]. No studies have showed a survival disadvantage based on race specifically for pRCC after adjusting for confounders.

An additional limitation of this study is limited gene-level mutation data for black (n=41) and white (n=30) patients. Compared to previous reports of a 12% mutation rate for MET, MET in the current study was not one of the most commonly mutated genes included for analysis, thus demonstrating the underestimation of MET mutations resulting from a low sample size [11–12]. A limitation of this study is that pRCC TCGA data is based on primary nephrectomy specimens [39, 40] and may limit the ability of these findings to be applied to predict response to metastatic pRCC treatments for black and white patients since primary metastatic vs. primary nephrectomy RCC specimens have significantly different expression profiles [41].

A primary confounding factor to our study is intra-tumor heterogeneity since the TCGA relies on a single-site sample of each specimen for sequencing [42]. Specifically distinct subclones with distinct mutation and expression within the same tumor may have provided us with different outcomes depending on the sample of the tumor sequenced. It is also unknown whether increased representation of CpG island methylator phenotype tumors which yield worse outcomes [43] were also overrepresented in black patients. This should be explored in future studies as this may help to further explain racial disparities in RCC.

We report the first study to compare the genomic landscape between black and white patients with pRCC. Distinct tumor biology was identified with differential expression of 283 genes and enrichment of the VEGF pathway, immune system pathways and overexpression of CRYBB2 associated with the WNT pathway in black patients. Thus, it is likely inaccurate to apply results from RCC biomarker and targeted therapy studies of predominantly white patients to the underrepresented black population. Our data requires validation and further elucidation in pre-clinical models, but these results may predict an increased immune response to the tumor in black patients and also an increased response to immunotherapies and or VEGFR inhibitors for black patients with advanced pRCC. Future studies and personalized medicine approaches relying on combination or sole forms of therapy with the use of immunotherapies and targeted therapies (VEGFR) may serve as a route to improve survival in black patients with pRCC. This should be explored in pre-clinical models and in clinical trials in the first-line or adjuvant setting for advanced and high-risk localized pRCC.

## MATERIALS AND METHODS

The present retrospective study relied on the University of California Santa Cruz Genome (UCSC) Browser to download publicly available gene-level non-silent somatic mutation data (nonsense, missense, frame-shift indels, splice site mutations, stop codon read-throught, change of start codon, inframe indels) relied on Firewbrowse to download RNASeq gene expression and clinical pRCC TCGA data [44]. The non-silent somatic mutation data used is data that the UCSC genome browser downloaded directly from the TCGA's data coordinating center and has processed at UCSC into their data repository. Calls for the broad curated mutation data were generated at the Broad Institute Genome Sequencing Center using the MutDect method. Clinical data and raw read count RNASeq expression data is direct and original TCGA data that Firebrowse (a portal of archived TCGA data hosted by the Broad Institute of MIT and Harvard) archives for public access and download.

Patients were excluded from the study if they had the following criteria: Asian or American Indian/Alaskan Native race, no race data available, metastatic disease, missing gene expression data, missing pathologic stage data. We included 254 (58 black, 196 white) of 291 patients with an initial diagnosis of pRCC from 2001-2013.

To reduce the confounding influence of selection bias in our gene-level analyses between black and white patients, we applied nearest-neighbor 1 to 1 propensity score matching on the cohort. While the use of a 1 to 1 vs. 2 to 1 propensity score matching ratio may result in a minor decrease in power and precision, this ratio results in less bias [45]. Patients were matched on age and gender in addition to pathologic T stage. Propensity score matching was conducted using the MatchIt package in R Studio version 0.98.1091 [46].

For the propensity matched cohort of 58 black and 58 white patients, we utilized the SAMseq package in R and performed significance analysis on microarray analysis on raw read count RNA-Seq gene expression data of 20,530 genes using 1000 repeated permutations and false discovery rate correction (q<.05).

In a subset of propensity matched pRCC patients with gene-level mutation and paired clinical data available (n=71), rates of non-silent somatic gene-level mutations were compared between 41 (57.7%) black and 30 (42.3%) white patients. Genes compared included genes identified as the most top 10 most frequently mutated in the dataset (i.e., abParts, DDX12P, FJL36000, FRG1B, CROCCP2, MUC4, MGC70870, MST1P2, PCDHGC5, TTN). Somatic mutation rates were compared using chi-squared tests of independence and fisher's exact tests.

For the overall cohort and for the propensity matched cohort, age, gender, BMI, Type 1 vs. Type 2 pRCC, clinical and pathologic TNM stages were compared between black and white patients. Also compared between groups were year of initial pathologic diagnosis, history of malignancy, history of neoadjuvant therapy, history of smoking, targeted molecular therapy use and use of adjuvant radiation therapy. Pathologic and clinical stage were treated in an ordinal fashion for analysis (i.e., T1=1, T2=2, T3=3, T4=4) since a, b and c sub-classification within staging was not available for all patients.

Overall survival (OS) and cancer specific survival (CSS) were compared in univariable cox proportion hazards models. Death was defined as related to renal cancer according to a previously defined method if the patient had clinical M1, a tumor present at the time of death or if composite tumor status was unavailable [47, 48].

All other continuous and categorical variables were compared using Mann-Whitney U tests and chi-squared tests of independence respectively. All statistical analysis was conducted using R Studio version 3.1.3 and SPSS Version 20.0.

Gene Set Enrichment Analysis (GSEA) [49] to evaluate differential activity of pathways and gene sets in white and black patient groups. Changes in gene expression between the two patient groups were evaluated using a t-statistic. 1077 gene sets from MSigDB v. 5.1 (using the c2 “curated pathways”, c6, and “Hallmark” collections) were evaluated for differential expression. To account for gene-gene correlations in the enrichment analysis, GSEA *P*-values were computed with respect to a null distribution obtained from 1000 randomizations of the patient-phenotype labels, and false discovery rates were estimated by the GSEA software.

## SUPPLEMENTARY MATERIALS FIGURES AND TABLES






