# Supplementary material for: Genomic differences between black and white patients implicate a distinct immune response to papillary renal cell carcinoma
Source: Oncotarget. 2016 Dec 23;8(3):5196–205. doi: 10.18632/oncotarget.14122 (PMC5354901; doi:10.18632/oncotarget.14122)
Supplement: Supplementary file 1 [file oncotarget-08-5196-s001.pdf]

Genomic differences between black and white patients implicate a distinct immune response to papillary renal cell carcinoma

SUPPLEMENTARY TABLES

Supplementary Table S1: All Genes Differentially Expressed between Black and White pRCC Patients.

See Supplementary File 1

Supplementary Table S2: All Genes Sets Identified from GSEA as Enriched in Black Patients (nominal p<0.100).

See Supplementary File 2
