# Supplementary material for: Genomic differences between black and white patients implicate a distinct immune response to papillary renal cell carcinoma
Source: Oncotarget. 2016 Dec 23;8(3):5196–205. doi: 10.18632/oncotarget.14122 (PMC5354901; doi:10.18632/oncotarget.14122)
Supplement: Supplementary file 2 [file oncotarget-08-5196-s002.docx]

|  |  |  |  |  |  |  |
| --- | --- | --- | --- | --- | --- | --- |
| **Supplemental Table 1. All Genes Differentially Expressed between Black and White pRCC Patients.** | | | | | | |
| **Black Patients** | | | | **White Patients** | | |
| **Gene Overexpressed** | | **q-value** | **Fold-Change** | **Gene Overexpressed** | **q-value** | **Fold-Change** |
| DHX40P1 | | 0.011 | 2.46E+08 | MAPK7 | 0.031 | 0.89 |
| ATCAY | | 0.031 | 2.46E+08 | PIGV | 0.044 | 0.88 |
| TREML4 | | 0.036 | 2.25E+08 | TOP3A | 0.014 | 0.88 |
| LOC100124692 | | 0.036 | 1.52E+08 | CDR2 | 0.023 | 0.87 |
| GSTM1 | | 0.05 | 42.23 | THUMPD1 | 0.027 | 0.87 |
| FCN2 | | 0 | 4.92 | DYNC1LI2 | 0.05 | 0.87 |
| GRIN2A | | 0.031 | 4.76 | ZC3H18 | 0.014 | 0.86 |
| FAM153A | | 0.043 | 4.66 | TAOK2 | 0.031 | 0.86 |
| UBD | | 0 | 4.38 | CCDC111 | 0.018 | 0.86 |
| CRYBB2 | | 0 | 4.37 | SC4MOL | 0.018 | 0.85 |
| FLT3 | | 0 | 3.93 | TMEM79 | 0.023 | 0.84 |
| FAM70A | | 0.018 | 3.41 | FAM86C | 0.027 | 0.84 |
| MGAM | | 0.05 | 3.35 | IFT140 | 0.014 | 0.83 |
| LRRC55 | | 0 | 3.33 | C1GALT1 | 0.044 | 0.83 |
| CCL3L1 | | 0.026 | 3.12 | WDR59 | 0 | 0.82 |
| SOX30 | | 0.018 | 2.84 | ABHD15 | 0.042 | 0.82 |
| JAKMIP1 | | 0.041 | 2.82 | SETD6 | 0.05 | 0.82 |
| GSTT2 | | 0.036 | 2.75 | IQCK | 0.023 | 0.82 |
| PRSS45 | | 0.031 | 2.57 | ELAC2 | 0.014 | 0.81 |
| GRAP2 | | 0.045 | 2.37 | MKS1 | 0.047 | 0.81 |
| EMR1 | | 0 | 2.34 | DEF8 | 0.027 | 0.81 |
| CA8 | | 0.031 | 2.28 | C7orf49 | 0.048 | 0.81 |
| CXCL9 | | 0.018 | 2.27 | TBCK | 0.032 | 0.81 |
| TARP | | 0.043 | 2.22 | ZNF747 | 0.042 | 0.8 |
| IRF4 | | 0.043 | 2.22 | DHX38 | 0.044 | 0.8 |
| CXCL10 | | 0.036 | 2.15 | CC2D2A | 0.014 | 0.8 |
| HLA-DPB2 | | 0.036 | 2.11 | CYB5B | 0.044 | 0.8 |
| CPT1C | | 0.05 | 2.11 | BTBD12 | 0.023 | 0.79 |
| P2RY10 | | 0.046 | 2.1 | DCAKD | 0 | 0.79 |
| LPL | | 0.05 | 2.08 | RNF40 | 0.014 | 0.79 |
| CHST1 | | 0 | 2.07 | C16orf53 | 0.048 | 0.79 |
| PDE2A | | 0 | 2.07 | TMEM143 | 0.018 | 0.79 |
| SCUBE1 | | 0.047 | 2.02 | TXNL4B | 0.014 | 0.79 |
| FAM162B | | 0.011 | 2.01 | RNF135 | 0 | 0.78 |
| GALNTL2 | | 0.021 | 1.98 | MRPS7 | 0 | 0.78 |
| SLC14A1 | | 0.018 | 1.98 | C16orf58 | 0.018 | 0.78 |
| NOTCH2NL | | 0 | 1.97 | c16orf88 | 0.014 | 0.77 |
| CXCL11 | | 0.036 | 1.97 | ADAL | 0.018 | 0.77 |
| PLXDC1 | | 0.036 | 1.96 | PPT2 | 0.048 | 0.77 |
| TMEM233 | | 0.021 | 1.95 | KIAA1609 | 0.023 | 0.76 |
| IFITM1 | | 0.038 | 1.94 | RPGRIP1L | 0.047 | 0.76 |
| LAMP3 | | 0.031 | 1.92 | HIRIP3 | 0.044 | 0.76 |
| RAB39B | | 0.031 | 1.91 | SIRPA | 0.014 | 0.75 |
| GATA2 | | 0.045 | 1.89 | CD2BP2 | 0.014 | 0.75 |
| CHST2 | | 0.018 | 1.87 | TELO2 | 0.014 | 0.74 |
| CSF2RB | | 0.045 | 1.86 | D2HGDH | 0.018 | 0.74 |
| C12orf59 | | 0.018 | 1.84 | CCDC103 | 0.018 | 0.74 |
| LOC441455 | | 0.021 | 1.83 | BCAR1 | 0.014 | 0.74 |
| TCTEX1D1 | | 0.045 | 1.82 | EXOSC6 | 0.014 | 0.73 |
| CETP | | 0.041 | 1.81 | IFT122 | 0 | 0.73 |
| ULK4 | | 0 | 1.81 | CCDC109A | 0.048 | 0.73 |
| LOC653113 | | 0.021 | 1.81 | FBF1 | 0.014 | 0.73 |
| GZMH | | 0.036 | 1.8 | TNFRSF12A | 0.036 | 0.73 |
| SERPINA5 | | 0.05 | 1.8 | C17orf100 | 0.047 | 0.73 |
| GZMA | | 0.036 | 1.78 | PMM2 | 0.018 | 0.72 |
| FGFBP2 | | 0.031 | 1.77 | LOC100129534 | 0.032 | 0.71 |
| GPIHBP1 | | 0 | 1.76 | C16orf71 | 0.023 | 0.71 |
| RHOH | | 0.036 | 1.75 | MGC45800 | 0.042 | 0.71 |
| CD40LG | | 0.045 | 1.75 | LEKR1 | 0 | 0.71 |
| PCDH12 | | 0 | 1.75 | HCG4 | 0.023 | 0.71 |
| RAB33A | | 0.045 | 1.75 | RPL23AP7 | 0 | 0.71 |
| ZBP1 | | 0.041 | 1.74 | COG4 | 0 | 0.7 |
| DUSP27 | | 0.046 | 1.71 | PPIL3 | 0 | 0.7 |
| EPHB3 | | 0.038 | 1.7 | CDK5R1 | 0.031 | 0.69 |
| GBP4 | | 0 | 1.7 | PEX11G | 0.027 | 0.69 |
| ROBO4 | | 0 | 1.69 | AMACR | 0.023 | 0.69 |
| RNF112 | | 0.031 | 1.69 | HSPA1B | 0.018 | 0.69 |
| GPR1 | | 0.036 | 1.67 | AXIN2 | 0.018 | 0.69 |
| IL3RA | | 0.031 | 1.67 | KDELR3 | 0 | 0.68 |
| GSTM3 | | 0 | 1.66 | NMRAL1 | 0.014 | 0.68 |
| NKG7 | | 0.043 | 1.64 | CTF1 | 0.018 | 0.67 |
| ST8SIA1 | | 0.05 | 1.64 | NUDT13 | 0 | 0.67 |
| PRF1 | | 0.036 | 1.64 | TMEM187 | 0 | 0.66 |
| P2RY8 | | 0.031 | 1.63 | CD320 | 0 | 0.65 |
| MCOLN2 | | 0.021 | 1.62 | FAM128A | 0.018 | 0.65 |
| ADAM19 | | 0.043 | 1.62 | ARL17A | 0 | 0.65 |
| EXOC3L2 | | 0.011 | 1.61 | GSTO2 | 0.014 | 0.64 |
| PLVAP | | 0.021 | 1.61 | APRT | 0 | 0.64 |
| ZNF521 | | 0.046 | 1.61 | LOC90784 | 0 | 0.64 |
| GIMAP6 | | 0.031 | 1.61 | LRRC37A | 0 | 0.63 |
| CD6 | | 0.038 | 1.6 | CABYR | 0.027 | 0.62 |
| CTLA4 | | 0.041 | 1.6 | ZNF117 | 0.018 | 0.62 |
| BCL6B | | 0.031 | 1.6 | FANCA | 0 | 0.62 |
| PLCXD1 | | 0.031 | 1.59 | LOC349196 | 0.014 | 0.62 |
| EMCN | | 0.011 | 1.59 | FUK | 0 | 0.61 |
| RGS5 | | 0.031 | 1.58 | FBXO15 | 0 | 0.6 |
| SH2D3C | | 0.018 | 1.58 | EP400NL | 0 | 0.6 |
| GIMAP4 | | 0.045 | 1.57 | TMEM139 | 0.047 | 0.6 |
| FLT4 | | 0.036 | 1.56 | C7orf13 | 0.014 | 0.6 |
| GIMAP7 | | 0.026 | 1.56 | LOC100133331 | 0.014 | 0.59 |
| KLHL5 | | 0.031 | 1.56 | TRAPPC2L | 0 | 0.59 |
| MMRN2 | | 0.047 | 1.55 | TUBB3 | 0.014 | 0.58 |
| GIMAP8 | | 0.036 | 1.55 | STON2 | 0 | 0.58 |
| C11orf21 | | 0.031 | 1.55 | PRODH | 0.036 | 0.57 |
| ETS1 | | 0.045 | 1.54 | RIBC2 | 0 | 0.53 |
| IL16 | | 0.038 | 1.54 | SHISA9 | 0.05 | 0.52 |
| GIMAP5 | | 0.021 | 1.54 | CCDC135 | 0.014 | 0.51 |
| P2RY14 | | 0.038 | 1.53 | SHH | 0.042 | 0.51 |
| KLRC1 | | 0.043 | 1.53 | SEC1 | 0 | 0.48 |
| EGFL7 | | 0.045 | 1.52 | LOC644165 | 0 | 0.48 |
| BLNK | | 0.031 | 1.52 | CN5H6.4 | 0 | 0.48 |
| ZNF93 | | 0.018 | 1.52 | MGC23270 | 0 | 0.47 |
| RET | | 0.045 | 1.51 | RDM1 | 0.027 | 0.47 |
| JPH4 | | 0.045 | 1.51 | C21orf56 | 0 | 0.45 |
| TMEM204 | | 0.031 | 1.5 | PKDREJ | 0 | 0.45 |
| GIPC3 | | 0.036 | 1.5 | SLC1A7 | 0.027 | 0.43 |
| PLAT | | 0.05 | 1.49 | BEGAIN | 0 | 0.42 |
| C3orf54 | | 0.031 | 1.48 | PCOLCE2 | 0 | 0.39 |
| ARHGEF15 | | 0 | 1.48 | LRRC37A2 | 0 | 0.39 |
| CIB2 | | 0.031 | 1.48 | DDX11L2 | 0.041 | 0.39 |
| ELTD1 | | 0.043 | 1.47 | AKR1C2 | 0.047 | 0.38 |
| ST6GALNAC3 | | 0.036 | 1.45 | HRNR | 0.023 | 0.37 |
| MYCT1 | | 0.05 | 1.45 | FAM90A1 | 0.027 | 0.34 |
| ICOSLG | | 0.046 | 1.45 | TMEM163 | 0 | 0.33 |
| PODXL | | 0.031 | 1.44 | LOC162632 | 0 | 0.32 |
| ADCY4 | | 0.011 | 1.43 | LQK1 | 0 | 0.31 |
| ADORA2A | | 0.021 | 1.42 | SYT17 | 0 | 0.29 |
| TIE1 | | 0.031 | 1.41 | CA9 | 0.048 | 0.2 |
| ERG | | 0.043 | 1.41 | UGT2A1 | 0.048 | 0.15 |
| TCF4 | | 0.045 | 1.41 | RPS28 | 0 | 0.04 |
| GJA4 | | 0.043 | 1.4 |  |  |  |
| PDGFB | | 0.038 | 1.4 |  |  |  |
| CCDC69 | | 0.045 | 1.38 |  |  |  |
| USHBP1 | | 0.045 | 1.38 |  |  |  |
| NLRP1 | | 0.045 | 1.37 |  |  |  |
| TSPAN7 | | 0.031 | 1.36 |  |  |  |
| SERHL | | 0.043 | 1.36 |  |  |  |
| C19orf63 | | 0.045 | 1.36 |  |  |  |
| EFCAB2 | | 0.038 | 1.36 |  |  |  |
| METTL10 | | 0 | 1.35 |  |  |  |
| CASP7 | | 0 | 1.35 |  |  |  |
| TAOK3 | | 0.036 | 1.33 |  |  |  |
| TRERF1 | | 0.045 | 1.32 |  |  |  |
| SNRK | | 0.045 | 1.31 |  |  |  |
| MX1 | | 0.031 | 1.31 |  |  |  |
| B3GALTL | | 0.038 | 1.31 |  |  |  |
| C10orf32 | | 0.036 | 1.3 |  |  |  |
| POLH | | 0.036 | 1.29 |  |  |  |
| GSTM4 | | 0.038 | 1.28 |  |  |  |
| DCLRE1C | | 0.031 | 1.27 |  |  |  |
| TRIM8 | | 0.018 | 1.27 |  |  |  |
| STK40 | | 0.043 | 1.26 |  |  |  |
| MGC2752 | | 0.021 | 1.26 |  |  |  |
| CHMP4C | | 0.031 | 1.25 |  |  |  |
| IFIH1 | | 0.041 | 1.24 |  |  |  |
| C12orf57 | | 0.041 | 1.24 |  |  |  |
| RAB12 | | 0.018 | 1.24 |  |  |  |
| OSTF1 | | 0.05 | 1.24 |  |  |  |
| PWP2 | | 0.031 | 1.22 |  |  |  |
| RSL24D1 | | 0.045 | 1.2 |  |  |  |
| SAP30L | | 0.038 | 1.2 |  |  |  |
| TMEM109 | | 0.05 | 1.2 |  |  |  |
| ATP6V0A2 | | 0.045 | 1.19 |  |  |  |
| C2orf3 | | 0.021 | 1.18 |  |  |  |
| C18orf10 | | 0.031 | 1.18 |  |  |  |
| C6orf62 | | 0.036 | 1.17 |  |  |  |
| ZNF85 | | 0 | 1.17 |  |  |  |
| ARMCX1 | | 0.045 | 1.16 |  |  |  |
| DPF2 | | 0.05 | 1.16 |  |  |  |
| AGPAT3 | | 0.031 | 1.15 |  |  |  |
| REPS1 | | 0.041 | 1.14 |  |  |  |
| MBIP | | 0.05 | 1.12 |  |  |  |
| C10orf46 | | 0.045 | 1.12 |  |  |  |
