# Supplementary material for: Genomic differences between black and white patients implicate a distinct immune response to papillary renal cell carcinoma
Source: Oncotarget. 2016 Dec 23;8(3):5196–205. doi: 10.18632/oncotarget.14122 (PMC5354901; doi:10.18632/oncotarget.14122)
Supplement: Supplementary file 3 [file oncotarget-08-5196-s003.docx]

| **Supplemental Table 2. All Genes Sets Identified from GSEA as Enriched in Black Patients (nominal p<0.100)** | | | | | | |
| --- | --- | --- | --- | --- | --- | --- |
| **NAME OF GENE SET** | **SIZE** | **ES** | **NES** | **NOM p-val** | **FDR q-val** | **FWER p-val** |
| BIOCARTA_NO1_PATHWAY | 30 | -0.70874184 | -1.9739596 | 0 | 0.2704501 | 0.091 |
| REACTOME_RAS_ACTIVATION_UOPN_CA2_INFUX_THROUGH_NMDA_RECEPTOR | 17 | -0.5920443 | -1.7734796 | 0.002024292 | 1 | 0.513 |
| REACTOME_ACTIVATION_OF_NMDA_RECEPTOR_UPON_GLUTAMATE_BINDING_AND_POSTSYNAPTIC_EVENTS | 37 | -0.5047599 | -1.7435285 | 0.003868472 | 0.8786992 | 0.59 |
| KEGG_CALCIUM_SIGNALING_PATHWAY | 177 | -0.42620632 | -1.6942835 | 0.003960396 | 0.8677167 | 0.708 |
| **REACTOME_CREB_PHOSPHORYLATION_THROUGH_THE_ACTIVATION_OF_CAMKII** | 15 | -0.61244696 | -1.7513416 | 0.00407332 | 1 | 0.566 |
| KEGG_LONG_TERM_POTENTIATION | 70 | -0.46491057 | -1.6636653 | 0.005758158 | 0.829491 | 0.76 |
| REACTOME_POST_NMDA_RECEPTOR_ACTIVATION_EVENTS | 33 | -0.5054749 | -1.6849285 | 0.005976096 | 0.7560029 | 0.728 |
| REACTOME_UNBLOCKING_OF_NMDA_RECEPTOR_GLUTAMATE_BINDING_AND_ACTIVATION | 15 | -0.64359 | -1.8150436 | 0.006198347 | 1 | 0.412 |
| BCAT_GDS748_UP | 48 | -0.4502103 | -1.5651703 | 0.01002004 | 1 | 0.873 |
| REACTOME_NUCLEOTIDE_LIKE_PURINERGIC_RECEPTORS | 16 | -0.6215002 | -1.6955792 | 0.010060363 | 0.9802352 | 0.707 |
| REACTOME_CREB_PHOSPHORYLATION_THROUGH_THE_ACTIVATION_OF_RAS | 27 | -0.4899514 | -1.5763243 | 0.014256619 | 1 | 0.864 |
| REACTOME_ACYL_CHAIN_REMODELLING_OF_PE | 21 | -0.532211 | -1.6530929 | 0.017716536 | 0.83226186 | 0.774 |
| KEGG_RENIN_ANGIOTENSIN_SYSTEM | 17 | -0.5732408 | -1.6861508 | 0.018556701 | 0.8332564 | 0.727 |
| REACTOME_CGMP_EFFECTS | 19 | -0.5516101 | -1.5997854 | 0.01996008 | 1 | 0.846 |
| KRAS.50_UP.V1_DN | 46 | -0.47917685 | -1.5743421 | 0.02008032 | 1 | 0.866 |
| NRL_DN.V1_DN | 129 | -0.3648123 | -1.4474478 | 0.025 | 1 | 0.945 |
| **PTEN_DN.V1_UP** | 185 | -0.33810085 | -1.4405639 | 0.025540275 | 1 | 0.95 |
| HALLMARK_KRAS_SIGNALING_DN | 198 | -0.34396556 | -1.4664253 | 0.027667984 | 1 | 0.931 |
| SINGH_KRAS_DEPENDENCY_SIGNATURE_ | 20 | -0.6633687 | -1.7199326 | 0.03137255 | 0.91468537 | 0.641 |
| REACTOME_NITRIC_OXIDE_STIMULATES_GUANYLATE_CYCLASE | 25 | -0.5191319 | -1.5428989 | 0.03420523 | 1 | 0.891 |
| REACTOME_INTERACTION_BETWEEN_L1_AND_ANKYRINS | 21 | -0.53994775 | -1.5525749 | 0.035019454 | 1 | 0.882 |
| KRAS.BREAST_UP.V1_DN | 137 | -0.33302554 | -1.4234372 | 0.03807615 | 1 | 0.954 |
| CTIP_DN.V1_UP | 126 | -0.37081912 | -1.4684211 | 0.03809524 | 1 | 0.93 |
| **NOTCH_DN.V1_UP** | 185 | -0.31978634 | -1.3947008 | 0.03992016 | 1 | 0.964 |
| CRX_NRL_DN.V1_DN | 126 | -0.35031307 | -1.3950937 | 0.040152963 | 1 | 0.964 |
| ATF2_S_UP.V1_UP | 188 | -0.29558066 | -1.3465997 | 0.040229887 | 1 | 0.975 |
| **BIOCARTA_VEGF_PATHWAY** | 29 | -0.51924455 | -1.4973836 | 0.0403071 | 1 | 0.918 |
| **REACTOME_DEFENSINS** | 40 | -0.5356761 | -1.582023 | 0.04192872 | 1 | 0.86 |
| PDGF_UP.V1_DN | 131 | -0.33081228 | -1.391168 | 0.045009784 | 1 | 0.965 |
| KEGG_TASTE_TRANSDUCTION | 51 | -0.45201316 | -1.5258429 | 0.04828974 | 1 | 0.905 |
| CAHOY_NEURONAL | 99 | -0.34128264 | -1.347266 | 0.048681542 | 1 | 0.975 |
| *REACTOME_G_ALPHA_S_SIGNALLING_EVENTS* | 120 | -0.3739809 | -1.4752142 | 0.051485147 | 1 | 0.923 |
| KRAS.600_UP.V1_UP | 273 | -0.32808176 | -1.3925245 | 0.05179283 | 1 | 0.964 |
| RELA_DN.V1_UP | 149 | -0.3560858 | -1.4759632 | 0.05222437 | 1 | 0.923 |
| **KEGG_NOD_LIKE_RECEPTOR_SIGNALING_PATHWAY** | 62 | -0.47515807 | -1.5150185 | 0.05421687 | 1 | 0.908 |
| REACTOME_ACYL_CHAIN_REMODELLING_OF_PI | 15 | -0.5142274 | -1.4575125 | 0.05719921 | 1 | 0.94 |
| REACTOME_TRANSPORT_OF_INORGANIC_CATIONS_ANIONS_AND_AMINO_ACIDS_OLIGOPEPTIDES | 93 | -0.35909218 | -1.3744324 | 0.058091287 | 1 | 0.973 |
| **IL2_UP.V1_DN** | 191 | -0.31070405 | -1.3185196 | 0.05836576 | 1 | 0.981 |
| REACTOME_SIGNALING_BY_NODAL | 18 | -0.51623666 | -1.5300514 | 0.05950096 | 1 | 0.902 |
| **KEGG_HEMATOPOIETIC_CELL_LINEAGE** | 84 | -0.4976166 | -1.5202769 | 0.06147541 | 1 | 0.907 |
| **IL21_UP.V1_UP** | 183 | -0.337084 | -1.4219561 | 0.06225681 | 1 | 0.956 |
| BIOCARTA_ERYTH_PATHWAY | 15 | -0.6221065 | -1.4618808 | 0.064794816 | 1 | 0.937 |
| REACTOME_NRAGE_SIGNALS_DEATH_THROUGH_JNK | 43 | -0.4697035 | -1.4719573 | 0.06876228 | 1 | 0.929 |
| REACTOME_ACYL_CHAIN_REMODELLING_OF_PS | 15 | -0.516322 | -1.4274144 | 0.06972112 | 1 | 0.954 |
| REACTOME_SYNTHESIS_OF_BILE_ACIDS_AND_BILE_SALTS | 19 | -0.47888538 | -1.416 | 0.07224335 | 1 | 0.958 |
| REACTOME_SIGNALING_BY_RHO_GTPASES | 109 | -0.4166483 | -1.4374744 | 0.076771654 | 1 | 0.95 |
| KEGG_GNRH_SIGNALING_PATHWAY | 100 | -0.3583223 | -1.3691931 | 0.07782101 | 1 | 0.973 |
| STK33_NOMO_DN | 271 | -0.3116718 | -1.3014969 | 0.07862903 | 1 | 0.982 |
| REACTOME_OTHER_SEMAPHORIN_INTERACTIONS | 15 | -0.5986036 | -1.5159632 | 0.08007449 | 1 | 0.908 |
| **HINATA_NFKB_IMMU_INF** | 17 | -0.6427212 | -1.5178707 | 0.08134921 | 1 | 0.907 |
| CYCLIN_D1_KE_.V1_DN | 190 | -0.32965988 | -1.3331013 | 0.08134921 | 1 | 0.978 |
| KEGG_ALDOSTERONE_REGULATED_SODIUM_REABSORPTION | 42 | -0.44720942 | -1.393044 | 0.086519115 | 1 | 0.964 |
| **REACTOME_BETA_DEFENSINS** | 34 | -0.47153425 | -1.3947291 | 0.08884297 | 1 | 0.964 |
| KRAS.300_UP.V1_UP | 143 | -0.31279638 | -1.2991823 | 0.09001957 | 1 | 0.982 |
| KEGG_NEUROACTIVE_LIGAND_RECEPTOR_INTERACTION | 271 | -0.34537032 | -1.3477505 | 0.09037328 | 1 | 0.975 |
| KEGG_GLYCOSPHINGOLIPID_BIOSYNTHESIS_GANGLIO_SERIES | 15 | -0.53759426 | -1.452682 | 0.09142857 | 1 | 0.944 |
| REACTOME_RAP1_SIGNALLING | 16 | -0.51984483 | -1.4276966 | 0.091796875 | 1 | 0.954 |
| REACTOME_EICOSANOID_LIGAND_BINDING_RECEPTORS | 15 | -0.5414779 | -1.4563762 | 0.09259259 | 1 | 0.941 |
| KRAS.300_UP.V1_DN | 136 | -0.3267049 | -1.3306384 | 0.09325397 | 1 | 0.978 |
| PRC2_EZH2_UP.V1_DN | 192 | -0.35134473 | -1.3395762 | 0.09542744 | 1 | 0.976 |
| REACTOME_SYNTHESIS_OF_PIPS_AT_THE_PLASMA_MEMBRANE | 30 | -0.5250201 | -1.4373003 | 0.09741551 | 1 | 0.95 |
| **KEGG_B_CELL_RECEPTOR_SIGNALING_PATHWAY** | 75 | -0.44780186 | -1.4269862 | 0.09741551 | 1 | 0.954 |
